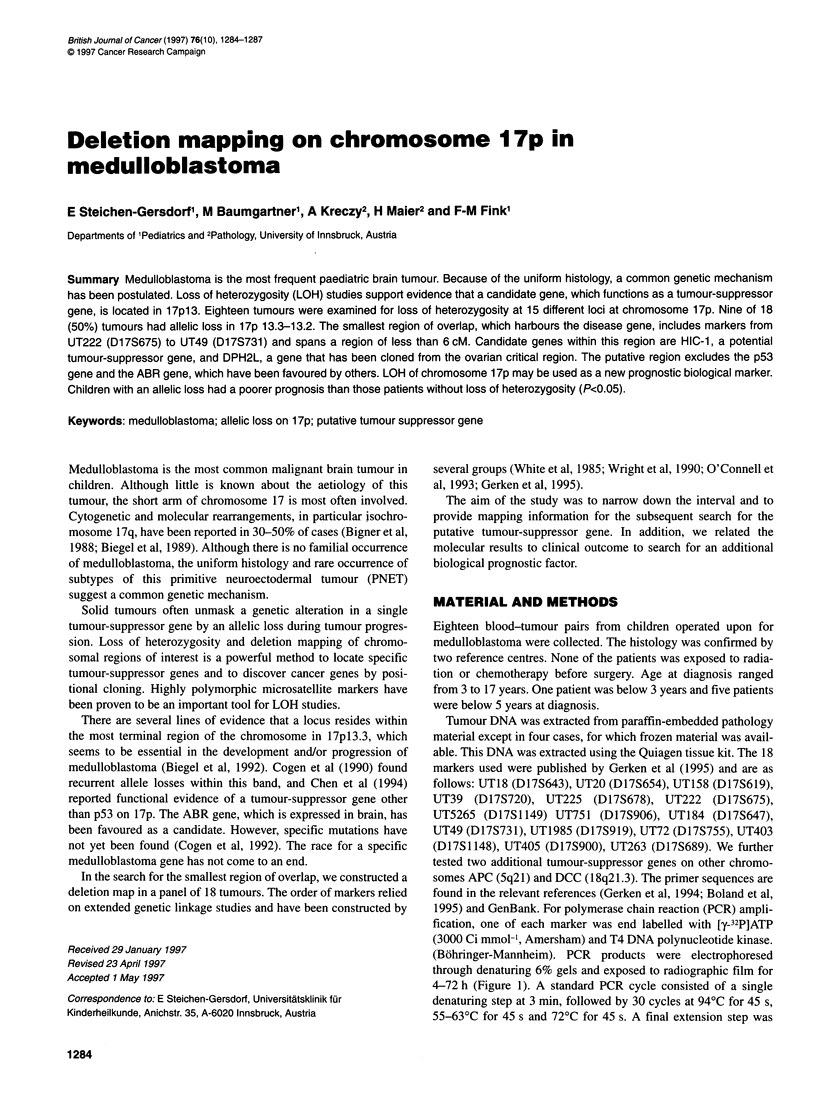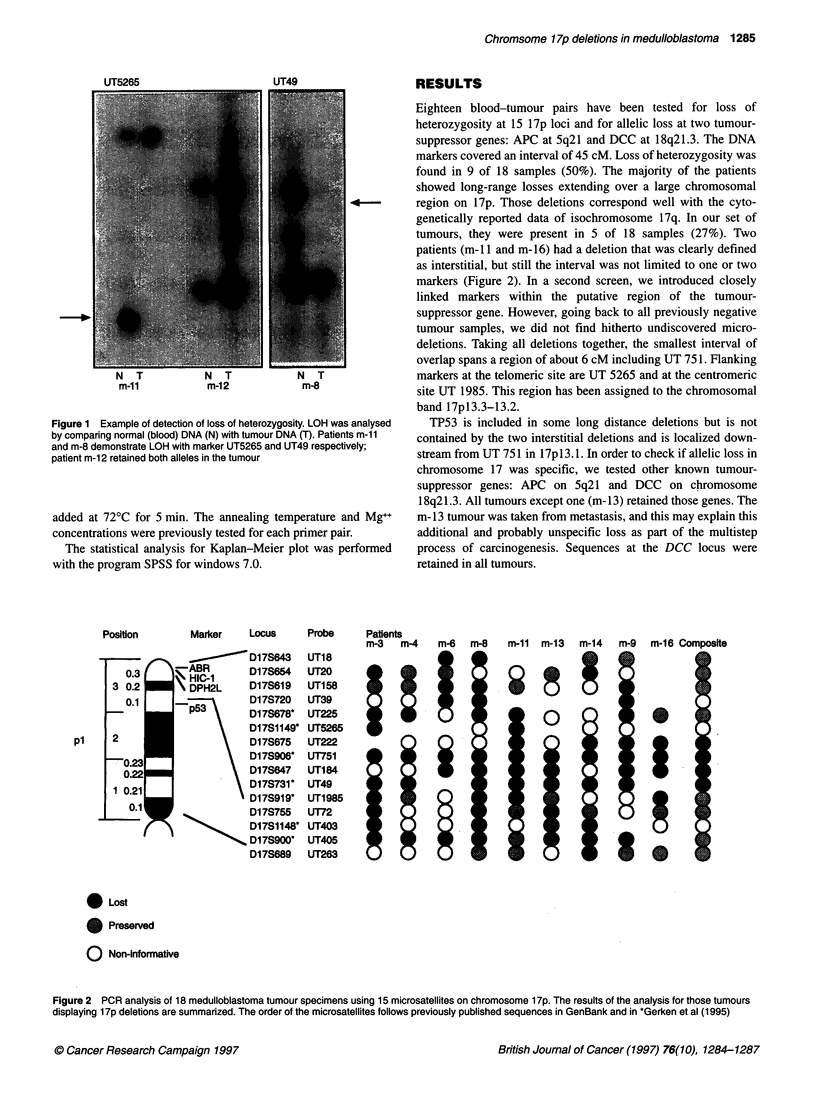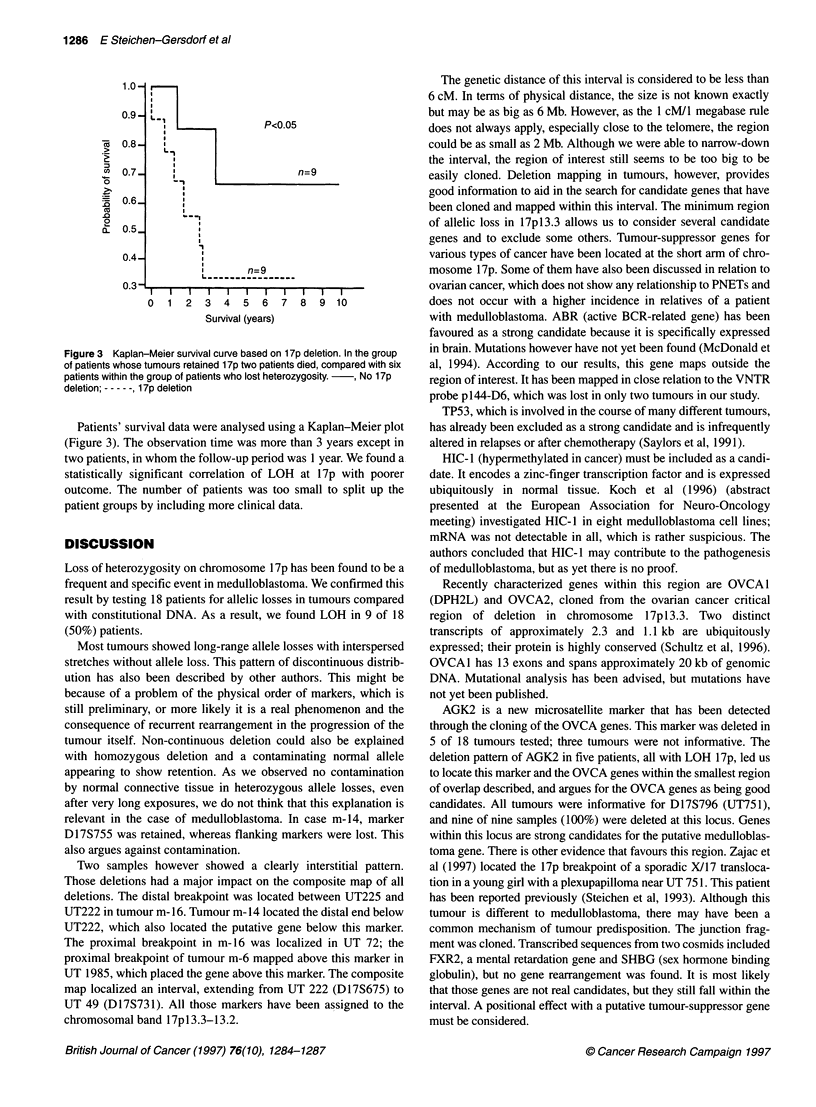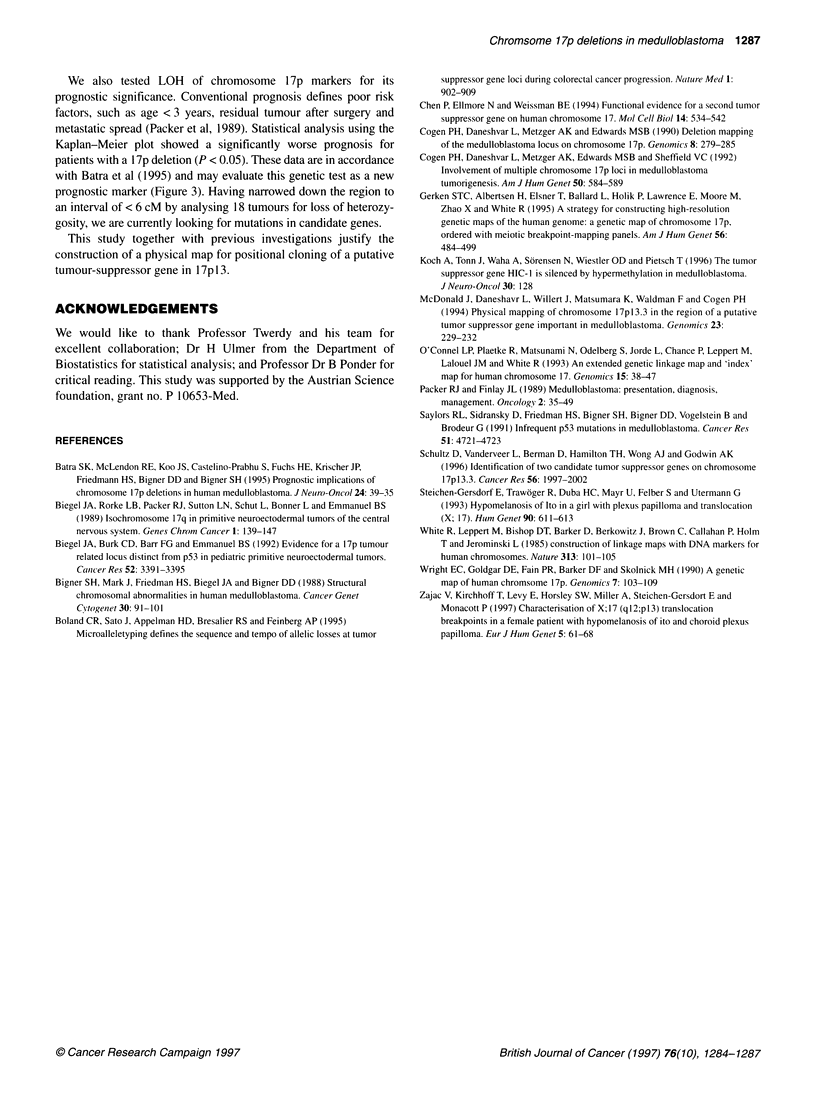# Deletion mapping on chromosome 17p in medulloblastoma.

**DOI:** 10.1038/bjc.1997.549

**Published:** 1997

**Authors:** E. Steichen-Gersdorf, M. Baumgartner, A. Kreczy, H. Maier, F. M. Fink

**Affiliations:** Department of Pediatrics, University of Innsbruck, Austria.

## Abstract

**Images:**


British Journal of Cancer (1997) 76(10), 1284–1287
© 1997 Cancer Research Campaign

### Deletion mapping on chromosome 17p in medulloblastoma

**E Steichen-Gersdorf[1], M Baumgartner[1], A Kreczy[2], H Maier[2] and F-M Fink[1]**

Departments of [1]Pediatrics and [2]Pathology, University of Innsbruck, Austria

**Summary** Medulloblastoma is the most frequent paediatric brain tumour. Because of the uniform histology, a common genetic mechanism has been postulated. Loss of heterozygosity (LOH) studies support evidence that a candidate gene, which functions as a tumour-suppressor gene, is located in 17p13. Eighteen tumours were examined for loss of heterozygosity at 15 different loci at chromosome 17p. Nine of 18 (50%) tumours had allelic loss in 17p 13.3–13.2. The smallest region of overlap, which harbours the disease gene, includes markers from UT222 (D17S675) to UT49 (D17S731) and spans a region of less than 6 cM. Candidate genes within this region are HIC-1, a potential tumour-suppressor gene, and DPH2L, a gene that has been cloned from the ovarian critical region. The putative region excludes the p53 gene and the ABR gene, which have been favoured by others. LOH of chromosome 17p may be used as a new prognostic biological marker. Children with an allelic loss had a poorer prognosis than those patients without loss of heterozygosity ($P<0.05$).

**Keywords**: medulloblastoma; allelic loss on 17p; putative tumour suppressor gene

Medulloblastoma is the most common malignant brain tumour in children. Although little is known about the aetiology of this tumour, the short arm of chromosome 17 is most often involved. Cytogenetic and molecular rearrangements, in particular isochromosome 17q, have been reported in 30–50% of cases (Bigner et al, 1988; Biegel et al, 1989). Although there is no familial occurrence of medulloblastoma, the uniform histology and rare occurrence of subtypes of this primitive neuroectodermal tumour (PNET) suggest a common genetic mechanism.

Solid tumours often unmask a genetic alteration in a single tumour-suppressor gene by an allelic loss during tumour progression. Loss of heterozygosity and deletion mapping of chromosomal regions of interest is a powerful method to locate specific tumour-suppressor genes and to discover cancer genes by positional cloning. Highly polymorphic microsatellite markers have been proven to be an important tool for LOH studies.

There are several lines of evidence that a locus resides within the most terminal region of the chromosome in 17p13.3, which seems to be essential in the development and/or progression of medulloblastoma (Biegel et al, 1992). Cogen et al (1990) found recurrent allele losses within this band, and Chen et al (1994) reported functional evidence of a tumour-suppressor gene other than p53 on 17p. The ABR gene, which is expressed in brain, has been favoured as a candidate. However, specific mutations have not yet been found (Cogen et al, 1992). The race for a specific medulloblastoma gene has not come to an end.

In the search for the smallest region of overlap, we constructed a deletion map in a panel of 18 tumours. The order of markers relied on extended genetic linkage studies and have been constructed by several groups (White et al, 1985; Wright et al, 1990; O'Connell et al, 1993; Gerken et al, 1995).

The aim of the study was to narrow down the interval and to provide mapping information for the subsequent search for the putative tumour-suppressor gene. In addition, we related the molecular results to clinical outcome to search for an additional biological prognostic factor.

### MATERIAL AND METHODS

Eighteen blood–tumour pairs from children operated upon for medulloblastoma were collected. The histology was confirmed by two reference centres. None of the patients was exposed to radiation or chemotherapy before surgery. Age at diagnosis ranged from 3 to 17 years. One patient was below 3 years and five patients were below 5 years at diagnosis.

Tumour DNA was extracted from paraffin-embedded pathology material except in four cases, for which frozen material was available. This DNA was extracted using the Quiagen tissue kit. The 18 markers used were published by Gerken et al (1995) and are as follows: UT18 (D17S643), UT20 (D17S654), UT158 (D17S619), UT39 (D17S720), UT225 (D17S678), UT222 (D17S675), UT5265 (D17S1149) UT751 (D17S906), UT184 (D17S647), UT49 (D17S731), UT1985 (D17S919), UT72 (D17S755), UT403 (D17S1148), UT405 (D17S900), UT263 (D17S689). We further tested two additional tumour-suppressor genes on other chromosomes APC (5q21) and DCC (18q21.3). The primer sequences are found in the relevant references (Gerken et al, 1994; Boland et al, 1995) and GenBank. For polymerase chain reaction (PCR) amplification, one of each marker was end labelled with [$\gamma$-$^{32}$P]ATP (3000 Ci mmol$^{-1}$, Amersham) and T4 DNA polynucleotide kinase. (Böhringer-Mannheim). PCR products were electrophoresed through denaturing 6% gels and exposed to radiographic film for 4–72 h (Figure 1). A standard PCR cycle consisted of a single denaturing step at 3 min, followed by 30 cycles at 94°C for 45 s, 55–63°C for 45 s and 72°C for 45 s. A final extension step was

*Received 29 January 1997*
*Revised 23 April 1997*
*Accepted 1 May 1997*

*Correspondence to:* E Steichen-Gersdorf, Universitätsklinik für Kinderheilkunde, Anichstr. 35, A-6020 Innsbruck, Austria

**Figure 1** Example of detection of loss of heterozygosity. LOH was analysed by comparing normal (blood) DNA (N) with tumour DNA (T). Patients m-11 and m-8 demonstrate LOH with marker UT5265 and UT49 respectively; patient m-12 retained both alleles in the tumour added at 72°C for 5 min. The annealing temperature and $Mg^{++}$ concentrations were previously tested for each primer pair.

The statistical analysis for Kaplan–Meier plot was performed with the program SPSS for windows 7.0.

### RESULTS

Eighteen blood–tumour pairs have been tested for loss of heterozygosity at 15 17p loci and for allelic loss at two tumour-suppressor genes: APC at 5q21 and DCC at 18q21.3. The DNA markers covered an interval of 45 cM. Loss of heterozygosity was found in 9 of 18 samples (50%). The majority of the patients showed long-range losses extending over a large chromosomal region on 17p. Those deletions correspond well with the cytogenetically reported data of isochromosome 17q. In our set of tumours, they were present in 5 of 18 samples (27%). Two patients (m-11 and m-16) had a deletion that was clearly defined as interstitial, but still the interval was not limited to one or two markers (Figure 2). In a second screen, we introduced closely linked markers within the putative region of the tumour-suppressor gene. However, going back to all previously negative tumour samples, we did not find hitherto undiscovered micro-deletions. Taking all deletions together, the smallest interval of overlap spans a region of about 6 cM including UT 751. Flanking markers at the telomeric site are UT 5265 and at the centromeric site UT 1985. This region has been assigned to the chromosomal band 17p13.3–13.2.

TP53 is included in some long distance deletions but is not contained by the two interstitial deletions and is localized downstream from UT 751 in 17p13.1. In order to check if allelic loss in chromosome 17 was specific, we tested other known tumour-suppressor genes: APC on 5q21 and DCC on chromosome 18q21.3. All tumours except one (m-13) retained those genes. The m-13 tumour was taken from metastasis, and this may explain this additional and probably unspecific loss as part of the multistep process of carcinogenesis. Sequences at the *DCC* locus were retained in all tumours.

**Figure 2** PCR analysis of 18 medulloblastoma tumour specimens using 15 microsatellites on chromosome 17p. The results of the analysis for those tumours displaying 17p deletions are summarized. The order of the microsatellites follows previously published sequences in GenBank and in *Gerken et al (1995)

© Cancer Research Campaign 1997

**Figure 3** Kaplan–Meier survival curve based on 17p deletion. In the group of patients whose tumours retained 17p two patients died, compared with six patients within the group of patients who lost heterozygosity. ——, No 17p deletion; - - - - -, 17p deletion

Patients' survival data were analysed using a Kaplan–Meier plot (Figure 3). The observation time was more than 3 years except in two patients, in whom the follow-up period was 1 year. We found a statistically significant correlation of LOH at 17p with poorer outcome. The number of patients was too small to split up the patient groups by including more clinical data.

### DISCUSSION

Loss of heterozygosity on chromosome 17p has been found to be a frequent and specific event in medulloblastoma. We confirmed this result by testing 18 patients for allelic losses in tumours compared with constitutional DNA. As a result, we found LOH in 9 of 18 (50%) patients.

Most tumours showed long-range allele losses with interspersed stretches without allele loss. This pattern of discontinuous distribution has also been described by other authors. This might be because of a problem of the physical order of markers, which is still preliminary, or more likely it is a real phenomenon and the consequence of recurrent rearrangement in the progression of the tumour itself. Non-continuous deletion could also be explained with homozygous deletion and a contaminating normal allele appearing to show retention. As we observed no contamination by normal connective tissue in heterozygous allele losses, even after very long exposures, we do not think that this explanation is relevant in the case of medulloblastoma. In case m-14, marker D17S755 was retained, whereas flanking markers were lost. This also argues against contamination.

Two samples however showed a clearly interstitial pattern. Those deletions had a major impact on the composite map of all deletions. The distal breakpoint was located between UT225 and UT222 in tumour m-16. Tumour m-14 located the distal end below UT222, which also located the putative gene below this marker. The proximal breakpoint in m-16 was localized in UT 72; the proximal breakpoint of tumour m-6 mapped above this marker in UT 1985, which placed the gene above this marker. The composite map localized an interval, extending from UT 222 (D17S675) to UT 49 (D17S731). All those markers have been assigned to the chromosomal band 17p13.3–13.2.

The genetic distance of this interval is considered to be less than 6 cM. In terms of physical distance, the size is not known exactly but may be as big as 6 Mb. However, as the 1 cM/1 megabase rule does not always apply, especially close to the telomere, the region could be as small as 2 Mb. Although we were able to narrow-down the interval, the region of interest still seems to be too big to be easily cloned. Deletion mapping in tumours, however, provides good information to aid in the search for candidate genes that have been cloned and mapped within this interval. The minimum region of allelic loss in 17p13.3 allows us to consider several candidate genes and to exclude some others. Tumour-suppressor genes for various types of cancer have been located at the short arm of chromosome 17p. Some of them have also been discussed in relation to ovarian cancer, which does not show any relationship to PNETs and does not occur with a higher incidence in relatives of a patient with medulloblastoma. ABR (active BCR-related gene) has been favoured as a strong candidate because it is specifically expressed in brain. Mutations however have not yet been found (McDonald et al, 1994). According to our results, this gene maps outside the region of interest. It has been mapped in close relation to the VNTR probe p144-D6, which was lost in only two tumours in our study.

TP53, which is involved in the course of many different tumours, has already been excluded as a strong candidate and is infrequently altered in relapses or after chemotherapy (Saylors et al, 1991).

HIC-1 (hypermethylated in cancer) must be included as a candidate. It encodes a zinc-finger transcription factor and is expressed ubiquitously in normal tissue. Koch et al (1996) (abstract presented at the European Association for Neuro-Oncology meeting) investigated HIC-1 in eight medulloblastoma cell lines; mRNA was not detectable in all, which is rather suspicious. The authors concluded that HIC-1 may contribute to the pathogenesis of medulloblastoma, but as yet there is no proof.

Recently characterized genes within this region are OVCA1 (DPH2L) and OVCA2, cloned from the ovarian cancer critical region of deletion in chromosome 17p13.3. Two distinct transcripts of approximately 2.3 and 1.1 kb are ubiquitously expressed; their protein is highly conserved (Schultz et al, 1996). OVCA1 has 13 exons and spans approximately 20 kb of genomic DNA. Mutational analysis has been advised, but mutations have not yet been published.

AGK2 is a new microsatellite marker that has been detected through the cloning of the OVCA genes. This marker was deleted in 5 of 18 tumours tested; three tumours were not informative. The deletion pattern of AGK2 in five patients, all with LOH 17p, led us to locate this marker and the OVCA genes within the smallest region of overlap described, and argues for the OVCA genes as being good candidates. All tumours were informative for D17S796 (UT751), and nine of nine samples (100%) were deleted at this locus. Genes within this locus are strong candidates for the putative medulloblastoma gene. There is other evidence that favours this region. Zajac et al (1997) located the 17p breakpoint of a sporadic X/17 translocation in a young girl with a plexupapilloma near UT 751. This patient has been reported previously (Steichen et al, 1993). Although this tumour is different to medulloblastoma, there may have been a common mechanism of tumour predisposition. The junction fragment was cloned. Transcribed sequences from two cosmids included FXR2, a mental retardation gene and SHBG (sex hormone binding globulin), but no gene rearrangement was found. It is most likely that those genes are not real candidates, but they still fall within the interval. A positional effect with a putative tumour-suppressor gene must be considered.

© Cancer Research Campaign 1997

We also tested LOH of chromosome 17p markers for its prognostic significance. Conventional prognosis defines poor risk factors, such as age < 3 years, residual tumour after surgery and metastatic spread (Packer et al, 1989). Statistical analysis using the Kaplan–Meier plot showed a significantly worse prognosis for patients with a 17p deletion ($P < 0.05$). These data are in accordance with Batra et al (1995) and may evaluate this genetic test as a new prognostic marker (Figure 3). Having narrowed down the region to an interval of < 6 cM by analysing 18 tumours for loss of heterozygosity, we are currently looking for mutations in candidate genes.

This study together with previous investigations justify the construction of a physical map for positional cloning of a putative tumour-suppressor gene in 17p13.

### ACKNOWLEDGEMENTS

We would like to thank Professor Twerdy and his team for excellent collaboration; Dr H Ulmer from the Department of Biostatistics for statistical analysis; and Professor Dr B Ponder for critical reading. This study was supported by the Austrian Science foundation, grant no. P 10653-Med.

### REFERENCES

Batra SK, McLendon RE, Koo JS, Castelino-Prabhu S, Fuchs HE, Krischer JP, Friedmann HS, Bigner DD and Bigner SH (1995) Prognostic implications of chromosome 17p deletions in human medulloblastoma. *J Neuro-Oncol* **24**: 39–35

Biegel JA, Rorke LB, Packer RJ, Sutton LN, Schut L, Bonner L and Emmanuel BS (1989) Isochromosome 17q in primitive neuroectodermal tumors of the central nervous system. *Genes Chrom Cancer* **1**: 139–147

Biegel JA, Burk CD, Barr FG and Emmanuel BS (1992) Evidence for a 17p tumour related locus distinct from p53 in pediatric primitive neuroectodermal tumors. *Cancer Res* **52**: 3391–3395

Bigner SH, Mark J, Friedman HS, Biegel JA and Bigner DD (1988) Structural chromosomal abnormalities in human medulloblastoma. *Cancer Genet Cytogenet* **30**: 91–101

Boland CR, Sato J, Appelman HD, Bresalier RS and Feinberg AP (1995) Microalleletyping defines the sequence and tempo of allelic losses at tumor suppressor gene loci during colorectal cancer progression. *Nature Med* **1**: 902–909

Chen P, Ellmore N and Weissman BE (1994) Functional evidence for a second tumor suppressor gene on human chromosome 17. *Mol Cell Biol* **14**: 534–542

Cogen PH, Daneshvar L, Metzger AK and Edwards MSB (1990) Deletion mapping of the medulloblastoma locus on chromosome 17p. *Genomics* **8**: 279–285

Cogen PH, Daneshvar L, Metzger AK, Edwards MSB and Sheffield VC (1992) Involvement of multiple chromosome 17p loci in medulloblastoma tumorigenesis. *Am J Hum Genet* **50**: 584–589

Gerken STC, Albertsen H, Elsner T, Ballard L, Holik P, Lawrence E, Moore M, Zhao X and White R (1995) A strategy for constructing high-resolution genetic maps of the human genome: a genetic map of chromosome 17p, ordered with meiotic breakpoint-mapping panels. *Am J Hum Genet* **56**: 484–499

Koch A, Tonn J, Waha A, Sörensen N, Wiestler OD and Pietsch T (1996) The tumor suppressor gene HIC-1 is silenced by hypermethylation in medulloblastoma. *J Neuro-Oncol* **30**: 128

McDonald J, Daneshavr L, Willert J, Matsumara K, Waldman F and Cogen PH (1994) Physical mapping of chromosome 17p13.3 in the region of a putative tumor suppressor gene important in medulloblastoma. *Genomics* **23**: 229–232

O'Connel LP, Plaetke R, Matsunami N, Odelberg S, Jorde L, Chance P, Leppert M, Lalouel JM and White R (1993) An extended genetic linkage map and 'index' map for human chromosome 17. *Genomics* **15**: 38–47

Packer RJ and Finlay JL (1989) Medulloblastoma: presentation, diagnosis, management. *Oncology* **2**: 35–49

Saylors RL, Sidransky D, Friedman HS, Bigner SH, Bigner DD, Vogelstein B and Brodeur G (1991) Infrequent p53 mutations in medulloblastoma. *Cancer Res* **51**: 4721–4723

Schultz D, Vanderveer L, Berman D, Hamilton TH, Wong AJ and Godwin AK (1996) Identification of two candidate tumor suppressor genes on chromosome 17p13.3. *Cancer Res* **56**: 1997–2002

Steichen-Gersdorf E, Trawöger R, Duba HC, Mayr U, Felber S and Utermann G (1993) Hypomelanosis of Ito in a girl with plexus papilloma and translocation (X; 17). *Hum Genet* **90**: 611–613

White R, Leppert M, Bishop DT, Barker D, Berkowitz J, Brown C, Callahan P, Holm T and Jerominski L (1985) construction of linkage maps with DNA markers for human chromosomes. *Nature* **313**: 101–105

Wright EC, Goldgar DE, Fain PR, Barker DF and Skolnick MH (1990) A genetic map of human chromsome 17p. *Genomics* **7**: 103–109

Zajac V, Kirchhoff T, Levy E, Horsley SW, Miller A, Steichen-Gersdort E and Monacott P (1997) Characterisation of X;17 (q12;p13) translocation breakpoints in a female patient with hypomelanosis of ito and choroid plexus papilloma. *Eur J Hum Genet* **5**: 61–68

© Cancer Research Campaign 1997